# Supplementary material for: Deletion of wfs1 Impairs Oligodendrocyte Precursor Cells Dorsal Distribution and Myelination Through the wfs1-hmgcs1 Axis in Zebrafish
Source: Biology (Basel). 2026 Aug 16;15(16):1402. doi: 10.3390/biology15161402 (PMC13510151; doi:10.3390/biology15161402)
Supplement: Supplementary file 1 [file biology-15-01402-s001.zip › File S1-Transcriptome sequencing data and differentially expressed genes (DEGs) in wfs1-- zebrafish at 5 dpf/1.GO/wfs1KOvsWT/ALL/wfs1KOvsWT.all.GOmf_DAG.pdf]

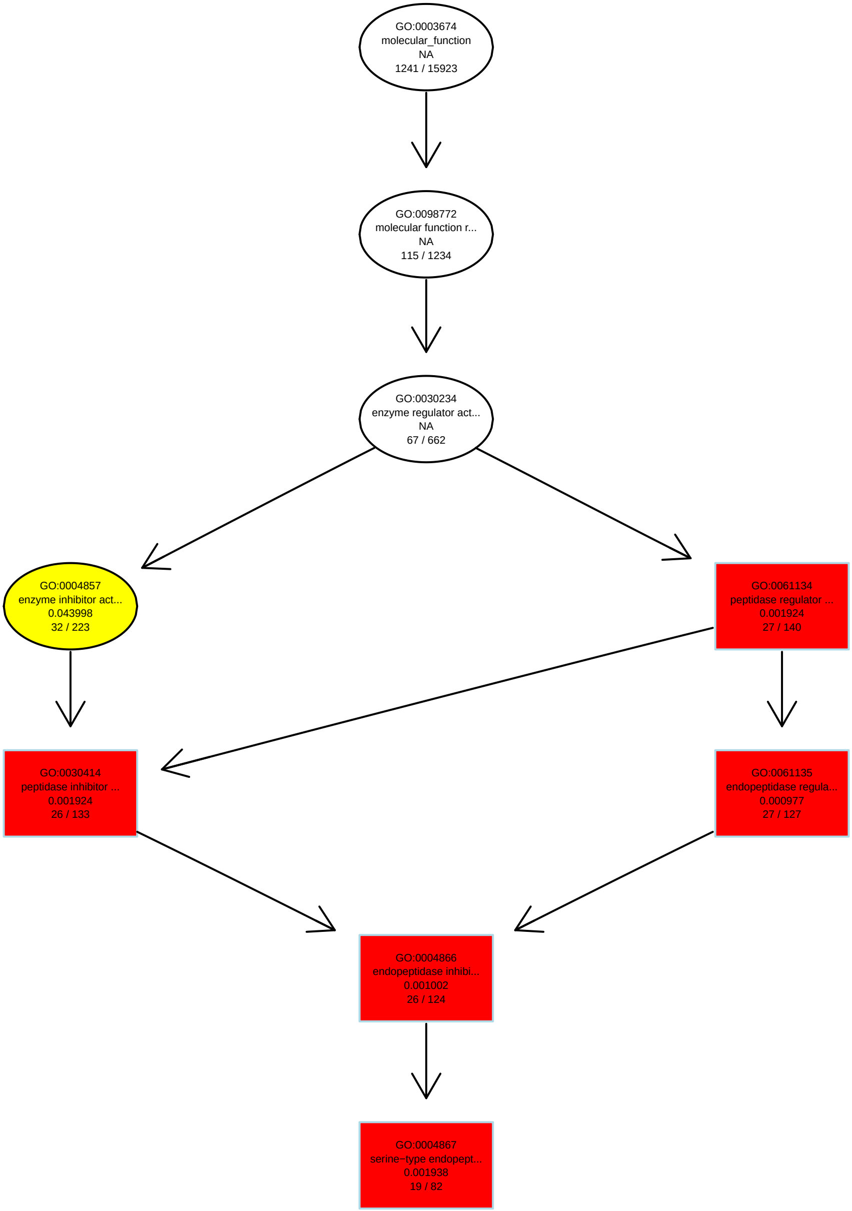
GO:0003674
molecular_function
NA
1241 / 15923
GO:0098772
molecular function r...
NA
115 / 1234
GO:0030234
enzyme regulator act...
NA
67 / 662
GO:0004857
enzyme inhibitor act...
0.043998
32 / 223
GO:0061134
peptidase regulator ...
0.001924
27 / 140
GO:0030414
peptidase inhibitor ...
0.001924
26 / 133
GO:0061135
endopeptidase regula...
0.000977
27 / 127
GO:0004866
endopeptidase inhibi...
0.001002
26 / 124
GO:0004867
serine−type endopept...
0.001938
19 / 82
